# Association of childhood socioeconomic status and health with depressive symptoms in later life: a cross-sectional latent class analysis of the 2014/2015 Indonesia Family Life Survey

**DOI:** 10.1136/bmjopen-2024-095197

**Published:** 2025-08-04

**Authors:** Ronny Isnuwardana, Jonathan Gibson, Asri Maharani, Herni Susanti, Helen Brooks, Penny Bee, Laura Anselmi

**Affiliations:** 1 Laboratory of Public Health and Community Medicine, Mulawarman University, Samarinda, East Kalimantan, Indonesia; 2 Division of Population Health, Health Services Research & Primary Care, The University of Manchester, Manchester, UK; 3 Mental Health Research Group, Division of Nursing, Midwifery and Social Work, School of Health Sciences, Faculty of Biology, Medicine, and Health and Manchester Academic Health Science Centre, The University of Manchester, Manchester, UK; 4 Faculty of Nursing, Universitas Indonesia, Depok, Jawa Barat, Indonesia

**Keywords:** EPIDEMIOLOGY, MENTAL HEALTH, Observational Study, Depression & mood disorders, PUBLIC HEALTH

## Abstract

**Abstract:**

**Objectives:**

While childhood circumstances predict mental health outcomes in high-income countries, evidence from low-income and middle-income countries (LMICs) like Indonesia remains scarce. This study examines the long-term association between childhood socioeconomic status (SES), health and depressive symptoms in adulthood, testing the hypothesis that early-life disadvantages increase the odds of depressive symptoms later in life.

**Design:**

Cross-sectional analysis using latent class analysis to cluster childhood SES/health and logistic regression to assess associations with depressive symptoms.

**Setting:**

A nationally representative household survey was conducted across 13 provinces in urban and rural areas of Indonesia.

**Participants:**

32 085 adults aged 18 years and older from the 2014–2015 Indonesia Family Life Survey. Participants with missing data on childhood circumstances or depressive symptoms were excluded, resulting in a final analytic sample of 29 140 individuals.

**Outcome measures:**

The primary outcome was depressive symptoms measured using the 10-item Centre for Epidemiologic Studies Depression Scale, with scores ≥10 indicating clinically significant symptoms. Secondary exposures included latent classes of childhood SES and health (high, moderate and low disadvantage). Analyses adjusted for adult SES, health behaviours, social capital and demographic characteristics.

**Results:**

Three latent classes emerged: low (64.85%), moderate (5.73%) and high (29.42%) early-life disadvantage. Adjusted logistic regression showed higher odds of depressive symptoms for high (OR 1.39, 95% CI 1.28 to 1.50) and moderate disadvantage (OR 1.66, 95% CI 1.48 to 1.87) versus low. Significant covariates included age, education, wealth and social capital (all p<0.05).

**Conclusions:**

Early-life disadvantages predict depressive symptoms in adulthood in Indonesia, underscoring the need for child-focused interventions (health, education and poverty reduction) to mitigate long-term mental health risks in LMICs. Further research should explore longitudinal mechanisms.

**STRENGTHS AND LIMITATIONS OF THIS STUDY**

- This study used a nationally representative sample, enhancing generalisability to the Indonesian adult population.
- This is the first study in Indonesia to examine life-course links between broad childhood conditions and adult depressive symptoms using robust clustering methods.
- Latent class analysis was applied to identify childhood socioeconomic and health condition subgroups based on multiple retrospective indicators.
- The cross-sectional design limits the causal interpretation of the associations observed.
- Key exposure variables were self-reported retrospectively, which may introduce recall bias and measurement error.

## Introduction

 Depression is among the leading contributors to mental health problems in the world, with a prevalence of around 3588.25 cases per 100 000 population and 46.87 million disability-adjusted life-years (DALYs) reported in 2019.^1^ The proportion of persons living with common mental health illnesses, such as depression and anxiety, in low-income and middle-income countries (LMICs) is increasing. Depression is projected to be among the third leading causes of disease burden in LMICs by 2030.^2^ Studies indicate that depression leads to substantial economic losses through reduced workplace productivity and increased absenteeism.^3^ The economic consequences of depression also include increased healthcare utilisation, further exacerbating the economic burden. On the other hand, depression can also affect physical health, potentially leading to conditions including cardiovascular disease and mortality.^4^ Depression also leads to a considerable number of suicides globally,^5^ heightening the importance of this disorder.

Indonesia is one of the LMICs, and to understand the magnitude of depression in Indonesia, it is important to describe the impact of this mental health condition. Indonesia has the second highest prevalence of depression and anxiety in the WHO South-East Asia region, with 14 million people living with these conditions.^6^ Across the age groups, a study by Sari *et al*^7^ indicated that over 3200 elementary to high school children in Indonesia were experiencing symptoms leading to mild to severe depressive disorders. Additionally, the Indonesia Basic Health Survey reported a 6.2% prevalence of depression among the population aged 15–24 years, highlighting the widespread impact of depression across different age groups in the country.

The impact of mental health problems has increased in Indonesia over the last decade, with a 22% increase in DALYs as a result of depressive disorders and an 18% increase in anxiety disorders. Research by Sinaga *et al*^8^ revealed that depression was linked to an increased risk of multimorbidity among the general population in Indonesia, emphasising the significant burden it places on individuals’ overall health and well-being. Putri *et al*^9^ found that depressive symptoms have economic implications due to potential productivity losses and healthcare costs associated with managing depression. This high burden is worsened by the limited mental healthcare access in LMICs, with only 7%–21% of people with depression and 5%–15% of people with anxiety receiving treatment.^10^ Therefore, identifying critical risk factors for depression is important for effective prevention and intervention strategies.

Significant risk factors for poor physical and mental health outcomes have been recognised, including exposure to adverse socioeconomic status (SES) and environmental conditions alongside pre-existing health issues, with childhood being a particularly critical period for development. Research has shown that lower childhood SES was associated with worse health outcomes and more depressive symptoms in later life, and conditions in childhood are significant predictors for later-life mental and physical health.^11^ Childhood factors impact later life health and adult social position, affecting health in middle age.^12^ Studies by Khanal and Selvamani ^13^ found that poor childhood health conditions and SES were risk factors for the emergence of depression in later life.

Related research also demonstrated that adverse early conditions and experiences increase risks across various pathways for depression. Adverse childhood experiences (ACEs), such as abuse, neglect and family conflict, are associated with an increased risk of depression in adulthood. Guerrero *et al*^14^ highlighted that ACEs, including trauma and distress, can increase the likelihood of developing depression. Furthermore, ACEs increase the likelihood of chronic health conditions, substance misuse and premature mortality in adulthood. Childhood maltreatment and peer victimisation are major risk factors for depression and suicidal behaviour, emphasising the long-lasting impact of ACEs on mental health in later life. Furthermore, research by Al-Shawi^15^ has shown that exposure to multiple risk factors during childhood, such as adverse experiences, is linked to higher rates of depression. These findings highlight the importance of early intervention and prevention efforts to address childhood conditions and promote positive mental health outcomes across the lifespan.

Understanding the role of these childhood influences is necessary for developing interventions and policies that mitigate negative effects in later life. This information can be captured either through longitudinal studies that follow up with individuals from childhood over an extended period to capture later life outcomes or retrospectively through surveys asking adults about their own childhood experiences. However, the main concern with retrospective data is that memories can fade with time and lead to incorrect recollections. This so-called recall bias introduces measurement error into the exposure variable.^16^

Recall bias is a common issue in studies that investigate childhood conditions and their impact on later life outcomes, such as depression. This bias occurs when participants are asked to recall events or experiences from their childhood, which can be influenced by their current mood, beliefs and experiences.^16^ For example, individuals who are currently experiencing depression may be more likely to recall negative experiences from their childhood, leading to an overestimation of the impact of childhood conditions on depression. Especially in the context of childhood conditions, such as childhood maltreatment or adverse experiences, retrospective cohort studies have been instrumental in comparing the associations between childhood adversity and negative outcomes using both retrospective and prospective assessments. Furthermore, when measuring childhood maltreatment to predict early adult psychopathology, studies have compared the use of prospective informant reports and retrospective self-reports. Both methods have been found to be valuable, although they come with potential limitations, such as underestimation and recall biases. Nonetheless, further measures can enhance the robustness of findings and account for recall bias inherent in retrospective self-reports.

Latent class analysis (LCA) was used to identify subgroups of individuals based on their patterns of responses across multiple retrospective indicators of childhood SES and health. This approach provides a classification that may be less sensitive to random errors in individual recall than single-measure analyses.^17^ Through the application of LCA, researchers can classify participants based on shared patterns in their responses to retrospective survey items, providing a structured approach to account for heterogeneity in reported childhood conditions. Therefore, this study aimed to evaluate the impact of childhood SES and health on later-life mental health in Indonesia using LCA applied to cross-sectional data from the 2014/2015 wave of the Indonesia Family Life Survey (IFLS).

## Methods

### Source and sample

We used data from the IFLS, a multifunctional longitudinal survey collecting information on individual and household demographics, SES and health characteristics from about 30 000 respondents from 12 000 households in 13 of 27 provinces in Indonesia.^18^ The IFLS was first administered in 1993, and four rounds of follow-up data collection occurred in 1997, 2000, 2007 and 2014. We used the fifth wave, which provided retrospective information on childhood conditions, enabling us to derive the primary predictor variable representing early-life SES and health through LCA. The IFLS Wave 5 sample contained 36 380 respondents. We limited our sample to 32 085 respondents aged 18 and over. We then excluded 2945 respondents who did not have any information on their childhood SES and health. The final sample size was 29 140 people (online supplemental figure 1). To assess the representativeness of the analytic sample and evaluate potential selection bias, we compared baseline characteristics between the total IFLS Wave 5 sample, the analytical sample (ie, respondents included in the final analysis), and those excluded due to missing data on key variables. These characteristics are presented in online supplemental table 1. Compared with the excluded group, the analytical sample had a higher proportion of females (53.4% vs 32.5%) and older average age (mean age 39.0 vs 35.3 years). Excluded participants were more likely to be single and less likely to be married. The review and approval for the IFLS were obtained from the institutional review boards in the USA (at RAND Corporation), and the Universitas Gadjah Mada in Indonesia, and informed consent was obtained from all study participants.

### Measurement of depressive symptoms

Depressive symptoms were assessed using the 10-item version of the Centre for Epidemiologic Studies Depression Scale (CES-D-10), a validated self-report instrument. Respondents were asked how often they experienced a range of symptoms during the past week, using a 4-point Likert scale (from ‘rarely or none of the time’ to ‘most of the time’). The two positively worded items were reverse-coded. Scores from the 10 items were summed, with higher scores indicating greater depressive symptom severity. Following established cut-offs, respondents with a total score of ≥10 were classified as having depressive symptoms.

### Childhood socioeconomic and health measures

We included three indicators of childhood SES (the availability of facilities in childhood homes, the number of books owned and overcrowding) and four indicators of childhood health (history of serious illnesses, absence from school a month or longer, hospitalisation for a month or longer and childhood hunger). Facilities indicated whether the respondent’s home had electricity, running water or indoor toilets at the age of 12. The available facilities ranged from 0 (no facilities) to 3 (all three facilities). The number of books in the house was the final measure to evaluate the childhood SES. We categorised the number of books into 0 for no or very few books (0–10 books) and 1 for enough books to fill one shelf or more. Overcrowding is classified as having more people than bedrooms in the childhood home. History of serious illnesses is self-reported and refers to having any of these diseases during childhood: infectious disease, polio, asthma, respiratory problems, allergies, severe diarrhoea, epilepsy, psychiatric problems, diabetes, heart diseases and malignancy. Respondents indicated if they were ever in hospital and missed school for 1 month or more because of a health condition during childhood. The following question was used to collect information on childhood hunger: “Did you ever experience hunger in your childhood (from birth to 15 years)?” Answers were recorded as yes or no.

### Confounders

In estimating the association between childhood SES and health and depressive symptoms, we controlled for an extensive set of covariates. Demographic characteristics include age and sex (male as reference). SES includes education, marital status, religion, employment status and expenditure per capita. We calculated household expenditure based on reported food and non-food expenditures, and per capita expenditure was constructed by dividing household expenditure by the number of persons in the household. Health conditions are captured using a set of self-reported binary indicators, including a family history of mental illness and whether the respondent has obesity, hypertension, diabetes, tuberculosis, asthma, chronic lung disease, chronic heart disease, liver disease, stroke, cancer, arthritis, hypercholesterolaemia, kidney disease, digestive disease, psychiatric disorders and memory-related disorders. Health behaviour included smoking status. We further included mobility, activity daily living (ADL) and instrumental ADL (IADL) to measure fundamental functional skills. To account for potential genetic and familial influences on adult depression, we included self-reported family history of depressive disorders.

### Statistical analysis

We performed the analysis in two steps. In the first step, we derived a latent construct of childhood SES and health, indicating whether the respondents’ childhood was in good SES and health conditions. We had no prior expectations of specific group classification. We let the data determine the loadings or weights of each indicator and estimate the proportion of people who were in poverty and poor health during childhood. The IFLS employs a multistage stratified sampling design, with stratification by province and clustering at the community and household levels. To account for this complex design, we applied sampling weights in the LCA and used robust standard errors clustered at the primary sampling unit level in the analyses. The number of distinct groups identified and chosen for final analyses was dependent on various criteria: a lower Akaike’s information criterion (AIC) and Bayesian information criterion (BIC) with each class added to the model; an entropy value of at least 0.8, and at least 5% of the respondents contained within each class. Respondents were assigned to a group based on their highest probability of group membership.

In the second step, we used logistic regression models to investigate the association between group membership and depressive symptoms. We performed the analysis in three models. The first model (model 1) included our key predictor variable, latent classes of childhood SES and health, along with demographics (age and sex) and marital status. The second model (model 2) includes current SES (education, social class, wealth, income). The final model (model 3) included all those covariates plus health behaviours (smoking behaviours and physical activity). The sample in each model was reduced to respondents with a full set of observed covariates, and the sample weight was taken into account.

To address missing data in the covariates used in the regression analysis, we employed multiple imputation using chained equations. This approach assumes that data are missing at random, conditional on the observed variables. Given that Little’s MCAR test indicated the data were not missing completely at random (p<0.0001), we used imputation to minimise bias from complete case analysis. The imputation model included all variables used in the logistic regression models: childhood SES and health indicators, depression status and demographic and health-related covariates such as age, sex, education, wealth quintile, smoking status, obesity and presence of chronic diseases. We generated 20 imputed datasets, performed logistic regression on each, and combined the estimates using Rubin’s rules. All estimation is done in Stata V.17.

### Patient and public involvement

Neither patients nor the public were involved in this secondary data analysis.

## Results

We constructed three latent class models to investigate childhood SES and early health variables. Table 1 summarises the class sizes and model fit statistics. The first model identified two latent classes: high (34.12%, n=9945) and low early life disadvantage (65.88%, n=19 204). The second model introduced three classes: high (29.41%, n=8573), moderate (5.73%, n=1669) and low disadvantage (64.86%, n=18 907). The third model further divided the sample into four classes: very high (1.15%, n=336), high (29.84%, n=8698), moderate (8.76%, n=2552) and low disadvantage (60.25%, n=17 563).

**Table 1:** Latent class analysis for the childhood socioeconomic status and early health variables

Latent class models	Class percentage				AIC	BIC	Entropy
	1	2	3	4			
Two-class model	34.12	65.88			177 164.833	177 289.029	0.387093
Three-class model	29.41	5.73	64.86		176 178.136	176 368.572	0.546366
Four-class model	1.15	29.84	8.75	60.25	176 034.436	176 291.110	0.558908

AIC, Akaike’s information criterion; BIC, Bayesian information criterion.

Although the four-class model showed slightly better goodness-of-fit metrics, with the lowest AIC (176 105.102) and BIC (176 361.786) values, its entropy was only marginally higher (0.5589 compared with 0.5463 in the second model). Additionally, the four-class model included a class comprising just 1.15% of the sample, reducing its interpretability. In contrast, the three-class model provided a good balance between model fit, classification accuracy and meaningful class proportions, with all classes exceeding 5% of the sample. Therefore, the three-class model was the most suitable for our analysis. Characteristics of the three classes, indicating high, moderate and low early life disadvantage, can be seen in table 2. The high early life disadvantage class was marked by substantial challenges, with a predominant majority reporting having no facilities (91.05%), none or very few books (97.77%) and overcrowding (87.60%). Moreover, this class reported notable health-related adversities, including a considerable percentage who experienced serious sickness during childhood (42.02%) and had ever been absent from school for a month or longer (12.50%). Respondents in the moderate early life disadvantage class had better childhood SES than those in the high early life disadvantage class. The highest proportion of them (37.99%) reported having one facility at their childhood home, followed by two (27.74%) and three facilities (23.07%). Lower proportions of them reported having none or very few books (75.61%) and overcrowding (80.83%). However, respondents in the moderate early life disadvantage class reported worse health than the other two. Most of them reported serious illness (84.48%) and being absent from school for a month or longer (94.61%). The low early life disadvantage class contrasts significantly, with lower proportions of respondents reporting challenges such as lower facilities scores (1.76%), limited access to books (74.95%) and high-density housing (75.67%) than the other two classes.

**Table 2:** Characteristics of observed variables between three-latent class, all values in frequency (percentages)

Variables	High early life disadvantage	Moderate early life disadvantage	Low early life disadvantage
	N=8571	N=1668	N=18 901
Had facilities score			
0 (less)	7804 (91.05)	187 (11.21)	332 (1.76)
1	489 (5.71)	634 (38.01)	6509 (34.44)
2	183 (2.14)	462 (27.70)	6063 (32.08)
3 (more)	94 (1.10)	385 (23.08)	5997 (31.73)
Had less books	8380 (97.77)	1262 (75.66)	14 166 (74.95)
Had high density house	7508 (87.60)	1348 (80.82)	14 301 (75.66)
Were sick during childhood	3601 (42.01)	1410 (84.53)	5737 (30.35)
Were ever absent from school ≥1 month	1072 (12.51)	1578 (94.60)	991 (5.24)
Were ever hospitalised ≥1 month	52 (0.61)	478 (28.66)	191 (1.01)
Were ever hungry	2106 (24.57)	222 (13.31)	126 (0.67)

Characteristics differences on demographic, adulthood SES and health behaviour between the three classes are reported in table 3. Respondents in the high early life disadvantage class are older (48.42±13.79) compared with those in the moderate early life disadvantage (36.55±11.99) and low early life disadvantage (34.94±12.45) classes. Those who experience high early life disadvantage tend to be employed, have lower educational attainment, be poorer and have comorbidities than those who do not.

**Table 3:** Characteristics of covariates between three-latent classes (N=28 279)

Covariates	High early life disadvantage	Moderate early life disadvantage	Low early life disadvantage	P value	Missing
	N=8573	N=1669	N=18 907		N=2945
Age (years)	48.42±13.79	36.55±11.99	34.94±12.45	0.0001	50.67±21.58
Female	4368 (50.96)	828 (49.64)	10 373 (54.88)	<0.001	1245 (42.28)
Employed	6300 (73.50)	1218 (72.98)	13 088 (69.23)	<0.001	1572 (53.38)
Urban life	3949 (46.06)	1030 (61.71)	12 203 (64.55)	<0.001	1828 (62.07)
Marital status				<0.001	
Single	314 (3.66)	202 (12.11)	3524 (18.64)		431 (14.63)
Married	7039 (82.13)	1368 (82.01)	14 291 (75.61)		1735 (58.91)
Separated	296 (3.45)	50 (3.00)	449 (2.38)		128 (4.35)
Widower	922 (10.76)	48 (2.88)	637 (3.37)		651 (22.11)
Education				<0.001	
Primary or lower	4765 (62.18)	492 (29.73)	3900 (20.96)		1022 (34.70)
Secondary	2505 (32.69)	929 (56.13)	11 003 (59.12)		1024 (34.77)
College	393 (5.13)	234 (14.14)	3708 (19.92)		313 (10.63)
Religion				<0.001	
Islam	7576 (88.38)	1500 (89.87)	17 125 (90.58)		152 (5.16)
Others	996 (11.62)	169 (10.13)	1780 (9.42)		21 (0.71)
Wealth quintile				<0.001	
First (lowest)	2089 (25.61)	293 (18.58)	2970 (16.97)		651 (22.11)
Second	1853 (22.71)	327 (20.74)	3233 (18.47)		549 (18.64)
Third	1646 (20.18)	344 (21.81)	3490 (19.94)		492 (16.71)
Fourth	1461 (17.91)	310 (19.66)	3710 (21.20)		486 (16.50)
Fifth (highest)	1109 (13.59)	303 (19.21)	4098 (23.42)		488 (16.57)
Social capital (0–14)	2.10±1.87	2.13±1.96	1.92±1.86	0.0001	0.00±0.00
Smoking				<0.001	
Smoker	3106 (36.23)	618 (37.03)	5808 (30.72)		1069 (36.30)
Past smoker	538 (6.28)	92 (5.51)	766 (4.05)		243 (8.25)
Non-smoker	4929 (57.49)	959 (57.46)	12 332 (65.23)		1439 (48.86)
Family history of mental illness	29 (0.34)	10 (0.60)	35 (0.19)	0.004	0 (0.00)
Obese	2708 (31.59)	520 (31.16)	6384 (33.77)	<0.001	251 (8.52)
Hypertension	1502 (17.52)	198 (11.86)	1958 (10.36)	<0.001	513 (17.42)
Diabetes mellitus	305 (3.56)	37 (2.22)	344 (1.82)	<0.001	92 (3.12)
Tuberculosis	83 (0.97)	28 (1.68)	164 (0.87)	0.004	41 (1.39)
Asthma	216 (2.52)	88 (5.27)	515 (2.72)	<0.001	94 (3.19)
Chronic lung disease	138 (1.61)	41 (2.46)	327 (1.73)	0.053	63 (2.14)
Heart disease	194 (2.26)	39 (2.34)	255 (1.35)	<0.001	64 (2.17)
Liver disease	73 (0.85)	35 (2.10)	191 (1.01)	<0.001	18 (0.61)
Stroke	92 (1.07)	12 (0.72)	82 (0.43)	<0.001	123 (4.18)
Cancer	61 (0.71)	19 (1.14)	112 (0.59)	0.023	16 (0.54)
Arthritis	756 (8.82)	98 (5.87)	653 (3.45)	<0.001	218 (7.40)
Hypercholesterolaemia	412 (4.81)	70 (4.19)	804 (4.25)	0.107	107 (3.63)
Kidney disease	155 (1.81)	28 (1.68)	230 (1.22)	0.001	38 (1.29)
Digestive disease	911 (10.63)	289 (17.32)	2654 (14.04)	<0.001	242 (8.22)
Psychiatric disorder	11 (0.13)	7 (0.42)	25 (0.13)	0.012	50 (1.70)
Memory-related disorder	15 (0.17)	7 (0.42)	25 (0.13)	0.018	39 (1.32)
Total mobility score (0–11)	0.30±0.85	0.18±0.58	0.16±0.55	0.0001	1.30±2.57
Total activity daily living score (0–5)	0.02±0.20	0.01±0.12	0.01±0.12	0.0002	0.29±0.99
Total instrumental activity daily living score (0–6)	0.24±0.8	0.20±0.67	0.16±0.61	0.0001	1.26±2.05

All values described as frequency (percentage) except age, social capital, total mobility score, total activity daily living score and total instrumental activity daily living score described as mean±SD. Hypothesis tests were conducted using Pearson χ2 test, except for variables age, social capital, total mobility score, total activity daily living score and total instrumental activity daily living score using Kruskal-Wallis’s test.

Finally, table 4 presents the results of multivariable logistic regression analyses assessing the relationship between latent classes and depressive symptoms outcome, while adjusting for relevant covariates across three models. The high early life disadvantage and moderate early life disadvantage classes exhibit consistently higher adjusted ORs for depressive symptoms across all three models compared with the reference low early life disadvantage class. In model 1, the high early life disadvantage class shows an OR of 1.48 (95% CI 1.37 to 1.60), which slightly decreases to 1.36 (95% CI 1.25 to 1.48) in model 2, and then to 1.36 (95% CI 1.24 to 1.48) in model 3. Similarly, the moderate early life disadvantage class demonstrates ORs of 1.90 (95% CI 1.68 to 2.14), 1.82 (95% CI 1.60 to 2.06), and 1.66 (95% CI 1.46 to 1.89) in models 1, 2 and 3, respectively. The covariates also contribute significantly to the depressive symptoms outcome. Notably, older age is associated with decreased odds of depressive symptoms in all models, while being female, employed and having lower education levels are associated with increased odds. Various health conditions such as current smoker, family history of mental illness, hypertension, asthma, chronic lung disease, chronic heart disease, liver disease, stroke, arthritis and digestive disease are also positively associated with depressive symptoms. A family history of mental illness was significantly associated with higher odds of depression (OR 2.02, 95% CI 1.16 to 3.51). Furthermore, increased scores on social capital, total mobility and total IADL are linked to higher odds of depressive symptoms.

**Table 4:** Multivariable logistic regression of depression outcome with class and covariates, showing adjusted OR (lower; upper 95% CI)

Variables	Model 1	Model 2	Model 3
High early life disadvantage class	1.48 (1.37 to 1.60)	1.36 (1.25 to 1.48)	1.36 (1.24 to 1.48)
Moderate early life disadvantage class	1.90 (1.68 to 2.14)	1.82 (1.60 to 2.06)	1.66 (1.46 to 1.89)
Age (years)	0.98 (0.97 to 0.98)	0.98 (0.97 to 0.98)	0.97 (0.97 to 0.97)
Female	1.03 (0.97 to 1.10)	1.10 (1.02 to 1.19)	1.36 (1.22 to 1.53)
Employed		1.10 (1.02 to 1.19)	1.15 (1.06 to 1.24)
Urban life		1.15 (1.08 to 1.23)	1.18 (1.10 to 1.26)
Marital status			
Single		1.50 (1.36 to 1.66)	1.44 (1.30 to1.60)
Married		reference	reference
Separated		1.70 (1.41 to 2.04)	1.61 (1.34 to 1.94)
Widower		1.33 (1.12 to 1.57)	1.25 (1.05 to 1.49)
Education			
Primary and lower		1.48 (1.31 to 1.67)	1.44 (1.27 to 1.63)
Secondary		1.18 (1.06 to 1.30)	1.15 (1.03 to 1.28)
College		reference	reference
Religion			
Islam		reference	reference
Others		1.05 (0.94 to 1.17)	1.04 (0.93 to 1.16)
Wealth quintile			
First (lowest)		1.16 (1.05 to 1.30)	1.19 (1.07 to 1.33)
Second		1.02 (0.92 to 1.14)	1.04 (0.93 to 1.16)
Third		1.03 (0.93 to 1.15)	1.05 (0.95 to 1.17)
Fourth		1.05 (0.94 to 1.16)	1.05 (0.95 to 1.17)
Fifth (highest)		reference	reference
Social capital		1.04 (1.02 to 1.05)	1.03 (1.02 to 1.05)
Smoking			
Smoker			1.43 (1.28 to 1.61)
Past smoker			1.14 (0.94 to 1.37)
Non-smoker			reference
Family history of mental illness			2.02 (1.16 to 3.51)
Obese			0.93 (0.87 to 1.00)
Hypertension			1.34 (1.21 to 1.49)
Diabetes mellitus			0.89 (0.70 to 1.12)
Tuberculosis			1.35 (0.98 to 1.87)
Asthma			1.44 (1.19 to 1.74)
Chronic lung disease			1.39 (1.10 to 1.76)
Chronic heart disease			1.32 (1.02 to 1.70)
Liver disease			1.44 (1.07 to 1.93)
Stroke			1.68 (1.12 to 2.53)
Cancer			1.37 (0.94 to 2.00)
Arthritis			1.69 (1.46 to 1.96)
Hypercholesterolaemia			1.07 (0.90 to 1.27)
Kidney disease			1.19 (0.92 to 1.55)
Digestive disease			1.49 (1.36 to 1.63)
Psychiatric disorder			1.63 (0.78 to 3.38)
Memory-related disorder			1.52 (0.80 to 2.91)
Total mobility score			1.18 (1.11 to 1.24)
Total activity daily living score			1.10 (0.87 to 1.40)
Total instrumental activity daily living score			1.13 (1.07 to 1.19)

The results of the logistic regression analyses after multiple imputation were consistent with those of the complete case analysis, reinforcing the robustness of our findings. The adjusted ORs were comparable across models (see online supplemental table 2).

## Discussion

We investigated the consequences of childhood SES and health on depressive symptoms in adulthood in Indonesia. Our results demonstrated that individuals classified as having high and moderate early life disadvantage had higher odds of being depressed than those experiencing low early life disadvantage. This is consistent with the wider literature in high-income countries, including New Zealand,^11^ USA^19^ and the UK.^20^ The impact of childhood SES on adult depression is also found in LMICs. Studies from India,^13^ Iran^21^ and South Africa^22^ have consistently demonstrated that individuals with lower SES during childhood are at a heightened risk of experiencing depression in adulthood. This risk is often compounded by factors such as ACEs, food insecurity and exposure to stressful life events.^22^ Research has highlighted that individuals with low childhood SES are more vulnerable to developing depressive symptoms as adults, with this risk being further exacerbated by experiences of childhood trauma.^23^

Research from various countries has consistently shown that the health status during childhood significantly impacts the risk for depression in adulthood. ACEs, including abuse, neglect and low SES, have been linked to an increased risk of mental health conditions, such as depression, throughout life.^24^ Studies have demonstrated that childhood SES influences the development and progression of depression in adulthood. Additionally, childhood health conditions and SES have been found to be important factors in diagnosing and preventing depressive symptoms in older adults.^25^ The long-term effects of childhood SES on mental health outcomes like depression extend into older age groups. Research has indicated that older individuals with a lower childhood SES are more likely to suffer from depression, emphasising the importance of addressing SES early in life to prevent adverse mental health outcomes in later years.^26^ Additionally, considering childhood SES and its impact on health is crucial for identifying significant factors associated with depression in older populations and implementing preventive measures.^13^ Furthermore, research has highlighted the long-term effects of ACEs on mental health outcomes, emphasising the importance of considering early-life conditions when assessing depression risk in middle and old age.^27^ Finally, childhood chronic physical illnesses have been linked to adult emotional problems, indicating a lasting impact of childhood health on emotional well-being.^28^

There are several plausible explanations for the association between childhood circumstances and depressive symptoms. The first likely explanation is through better perceived control and problem solving among individuals with better SES. Prior study showed that having a sense of control over one’s life and a supportive social environment with a higher SES can explain why some people have better mental health.^29^ On the other hand, those with a lower SES may face more obstacles in their lives, which can negatively affect their mental health by limiting their direction, control and planning in their life and work.^30^ Another explanation is that the association could be mediated by stress-related effects on neural circuitry, particularly in regions like the prefrontal cortex involved in reward processing.^31^ Additionally, childhood infections have been linked to depressive symptoms in later life, which may be due to impaired neurodevelopment caused by the interaction of infection, inflammation and metabolic factors.^32^ For instance, the cytokine interleukin-6, an inflammatory marker, has been associated with subsequent depressive symptoms.^33^

Our findings also suggest that social capital and a sense of control may be key mechanisms through which early life disadvantage translates into adult mental health outcomes. Individuals from low-SES backgrounds often have reduced access to supportive social networks and fewer opportunities to develop a sense of mastery or autonomy. This diminished social capital can limit their coping strategies and increase their vulnerability to depression. Prior research has shown that individuals with a strong sense of control and social support tend to experience fewer depressive symptoms, even in the face of economic or environmental adversity.^34 35^ Conversely, those with limited control over their life circumstances are more likely to experience chronic stress, which is a well-established pathway to depression.^35 36^ The lower social capital scores observed among respondents with high early life disadvantage in our study may, therefore, serve as both a marker and mediator of depression risk.

In addition, our results showed that functional limitations, specifically reduced mobility, are significantly associated with increased odds of depression. Mobility impairments can restrict daily activities, reduce social engagement and foster dependency, all of which can contribute to emotional distress and poor mental health.^37^ This finding aligns with existing literature that highlights the reciprocal relationship between physical disability and mental health.^38 39^ Not only can reduced mobility lead to isolation and a loss of autonomy, but depression itself can also exacerbate physical symptoms and limit motivation to remain active. Therefore, interventions that promote physical functioning and social participation may serve a dual role in mitigating depression, particularly among individuals with early-life disadvantages.

The significant association between family history of mental illness and adult depression aligns with established evidence on genetic and intergenerational transmission of mental health conditions.^40^ However, the very low prevalence of this variable (less than 0.5% of respondents) suggests that it may be underreported, potentially due to stigma, lack of diagnosis or cultural factors.^41^ Therefore, while the effect estimate is meaningful, it should be interpreted with caution. Future studies using clinically verified or more sensitively collected family history data would help clarify this relationship in the Indonesian context.

This study has several limitations. First, as an observational cross-sectional analysis, the results demonstrate associations rather than causal relationships. Second, the retrospective assessment of childhood SES and health indicators may be subject to recall bias, a limitation inherent to all survey studies using self-reported life course data. While LCA helped identify meaningful subgroups based on response patterns across multiple indicators, this methodological approach does not eliminate fundamental recall bias. Third, our outcome measure assessed depressive symptoms using the CES-D-10 scale rather than clinical diagnoses of depression.

Although the IFLS is nationally representative by design, differences between the analytic sample and those excluded due to missing data (as shown in online supplemental table 1) raise important considerations. The analytical sample had a higher proportion of older and female respondents and differed in marital status distribution. These differences may reflect patterns in who is more likely to provide complete data and may limit the generalisability of our findings to the broader population. We acknowledge this as a potential source of selection bias and have addressed it in part through multiple imputation for key variables, while also highlighting the issue as a limitation of the study.

These limitations are balanced by several strengths, including the use of a nationally representative sample that enhances generalisability to Indonesia’s adult population and the first to examine the life-course associations between childhood circumstances and adult depressive symptoms in this understudied context. In conclusion, we found that childhood SES and health significantly affect the risk of depressive symptoms during adulthood. Our findings emphasise the importance of the life course perspective in studying the impact of childhood circumstances on future mental health. Future research should prioritise identifying the specific pathways through which the impact is found. Our findings imply that more attention should be paid to investing in health capital in early life, with benefits extending throughout the entire lifetime.

## Supplementary material

10.1136/bmjopen-2024-095197 online supplemental file 1

## Data Availability

Data are available on reasonable request.
