# Supplementary material for: Association of childhood socioeconomic status and health with depressive symptoms in later life: a cross-sectional latent class analysis of the 2014/2015 Indonesia Family Life Survey
Source: BMJ Open. 2025 Aug 4;15(8):e095197. doi: 10.1136/bmjopen-2024-095197 (PMC12323512; doi:10.1136/bmjopen-2024-095197)
Supplement: online supplemental file 1 [file bmjopen-15-8-s001.docx]

**Supplementary Figure 1** Flow diagram of sample selection from IFLS5 respondents to final analytic sample with childhood data

**Supplementary Table 1** Baseline characteristics of the total sample, analytic sample, and excluded participants

|  | Included  N=29,140 | Excluded  N=7,240 | Total  N=36,380 |
| --- | --- | --- | --- |
| Female | 15,570 (53.43) | 2,355 (32.54) | 17,925 (49.21) |
| Age | 38.99 ± 14.20 | 35.26 ± 30.42 | 38.44 ± 17.65 |
| Marital status |  |  |  |
| Single | 4,040 (13.86) | 2,690 (37.17) | 6,730 (18.50) |
| Married | 22,693 (77.87) | 1,647 (23.13) | 24.367 (66.98) |
| Separated | 790 (2.71) | 131 (1.81) | 921 (2.53) |
| Widower | 1,613 (5.53) | 627 (8.66) | 2,240 (6.16) |
| Others | 13 (0.04) | 2,109 (29.17) | 2,122 (5.83) |

**Supplementary Table 2** Multivariable logistic regression with multiple imputation of depression outcome with class and covariates, showing adjusted odds ratio (lower; upper 95% confidence interval)

| Variables | Model 1 | Model 2 | Model 3 |
| --- | --- | --- | --- |
| High early life disadvantage class | 1.48 (1.37; 1.60) | 1.39 (1.29; 1.51) | 1.38 (1.27; 1.49) |
| Moderate early life disadvantage class | 1.90 (1.68; 2.14) | 1.89 (1.68; 2.14) | 1.75 (1.55; 1.98) |
| Age (years) | 0.98 (0.97; 0.98) | 0.98 (0.97; 0.98) | 0.97 (0.97; 0.97) |
| Female | 1.03 (0.97; 1.10) | 1.1 (1.03; 1.18) | 1.34 (1.20; 1.49) |
| Employed |  | 1.08 (1.00; 1.16) | 1.14 (1.06; 1.22) |
| Urban life |  | 1.15 (1.08; 1.23) | 1.17 (1.09; 1.25) |
| Marital status |  |  |  |
| Single |  | 1.52 (1.38; 1.67) | 1.46 (1.33; 1.61) |
| Married |  | reference | reference |
| Separated |  | 1.64 (1.38; 1.95) | 1.57 (1.32; 1.87) |
| Widower |  | 1.33 (1.14; 1.55) | 1.25 (1.07; 1.47) |
| Education |  |  |  |
| Primary and lower |  | 1.48 (1.32; 1.66) | 1.46 (1.3; 1.64) |
| Secondary |  | 1.17 (1.06; 1.29) | 1.14 (1.03; 1.26) |
| College |  | reference | reference |
| Religion |  |  |  |
| Islam |  | reference | reference |
| Others |  | 1.1 (0.99; 1.22) | 1.07 (0.96; 1.18) |
| Wealth quintile |  |  |  |
| 1^st^ (lowest) |  | 1.14 (1.02; 1.26) | 1.18 (1.06; 1.31) |
| 2^nd^ |  | 1.01 (0.91; 1.13) | 1.03 (0.93; 1.15) |
| 3^rd^ |  | 1.03 (0.93; 1.14) | 1.05 (0.95; 1.17) |
| 4^th^ |  | 1.04 (0.94; 1.15) | 1.05 (0.95; 1.17) |
| 5^th^ (highest) |  | reference | reference |
| Social capital |  | 1.04 (1.02; 1.06) | 1.04 (1.02; 1.06) |
| Smoking |  |  |  |
| Smoker |  |  | 1.4 (1.26; 1.56) |
| Past smoker |  |  | 1.09 (0.92; 1.30) |
| Non-smoker |  |  | reference |
| Family history of mental illness |  |  | 2.02 (1.18; 3.44) |
| Obese |  |  | 0.92 (0.85; 0.98) |
| Hypertension |  |  | 1.33 (1.20; 1.46) |
| Diabetes mellitus |  |  | 0.88 (0.70; 1.09) |
| Tuberculosis |  |  | 1.26 (0.92; 1.71) |
| Asthma |  |  | 1.43 (1.20; 1.70) |
| Chronic lung disease |  |  | 1.31 (1.05; 1.63) |
| Chronic heart disease |  |  | 1.31 (1.03; 1.66) |
| Liver disease |  |  | 1.44 (1.09; 1.90) |
| Stroke |  |  | 1.70 (1.17; 2.46) |
| Cancer |  |  | 1.38 (0.97; 1.98) |
| Arthritis |  |  | 1.69 (1.48; 1.94) |
| Hypercholesterolemia |  |  | 1.07 (0.92; 1.26) |
| Kidney disease |  |  | 1.30 (1.01; 1.66) |
| Digestive disease |  |  | 1.48 (1.36; 1.62) |
| Psychiatric disorder |  |  | 1.65 (0.84; 3.21) |
| Memory-related disorder |  |  | 1.28 (0.69; 2.37) |
| Total mobility score |  |  | 1.16 (1.10; 1.22) |
| Total activity daily living score |  |  | 1.18 (0.97; 1.44) |
| Total instrumental activity daily  living score |  |  | 1.12 (1.07; 1.18) |
