# Supplementary material for: Association of childhood socioeconomic status and health with depressive symptoms in later life: a cross-sectional latent class analysis of the 2014/2015 Indonesia Family Life Survey
Source: BMJ Open. 2025 Aug 4;15(8):e095197. doi: 10.1136/bmjopen-2024-095197 (PMC12323512; doi:10.1136/bmjopen-2024-095197)
Supplement: Supplementary file 2 [file bmjopen-2024-095197.reviewer_comments.pdf]

PEER REVIEW HISTORY

BMJ Open publishes all reviews undertaken for accepted manuscripts. Reviewers are asked to complete a checklist review form and are provided with free text boxes to elaborate on their assessment. These free text comments are reproduced below.

ARTICLE DETAILS

Title (Provisional)

Association of childhood socioeconomic status and health with depressive symptoms in later life: a cross-sectional latent class analysis of the 2014/2015 Indonesia Family Life Survey

Authors

Isnuwardana, Ronny; Gibson, Jonathan; Maharani, Asri; Susanti , Herni; Brooks, Helen; Bee, Penny; Anselmi, Laura

---

VERSION 1 - REVIEW

**Reviewer**	**1**
**Name**	**Selvamani, Y.**
**Affiliation**	**SRM Institute of Science and Technology, Public Health**
**Date**	**05-Jan-2025**
**COI**	**None**

This findings of the study suggest the significant association of childhood socioeconomic status with later life depression. However, the authors must address the grammatical issues. For ex.Among the covariates, age, education, 52 wealth quintile, social capital, and depression were associated with depression.

Further, In table 3, the social capital variable has few numbers. Similarly with other variables Total mobility score, Total activity daily living score and Total instrumental activity daily living score, the numbers are few

---

**Reviewer**	**2**
**Name**	**Mayer, Gwendolyn**
**Affiliation**	**University Hospital Heidelberg, Internal Medicine and Psychosomatics**
**Date**	**25-Feb-2025**
**COI**	**None**

Thank you for the opportunity to review this interesting work. The paper is well-written and structured. It presents data from a survey study that shows the association between socio-economic status and depressive symptoms using latent class analysis.

Few amendments will improve the overall readability and clarity:

Two general questions:

1. Was religion assessed in the survey? If yes, it should be part of the discussion, as it might impact the questions of depressive symptoms to a certain extent.

2. Though socio-economic influences are important to explain mental disorders, there is a big impact of heritability in the prevalence of depression. Did the survey ask for mental health of family members? If not, this limitation should be discussed.

Abstract: The objective focus on depressive symptom not on depression – this applies to several other sentences, as the CES-D 10 does not provide a clinical diagnosis.

Introduction: First sentence:

- the first source presents data on the increase of depression during COVID in 2020, either you should mention this or look for another, more generalizable source.

- What's more: the interpunctuation of the numbers is confusing 49.9 million can be a dot or a comma, depending on the journals formatting style, but should not be the same as 100.000 (indicating the thousand)

Line 79. Studies have shown that depression can significantly contribute to economic loss, affecting workplace productivity and absenteeism. A significant indirect cost related to productivity loss was also attributed to major depressive disorder [illegible] this seems a repetition, grammar should be checked

Line 150: the statement that LCA can reduce recall bias is a somewhat bold claim – LCA is just a statistical method not more. I understand the motivation but don't see that it is necessary to justify the choice in this way. No doubt, that the identification of subgroups is needed.

Line 156: facilitating a more precise analysis of recall bias – this is not the aim of this study. Recall bias is an assumption that might apply to the study population, but cannot be validated on the basis of this data.

Line 160: applied to longitudinal data – the data analysed was one of the waves of longitudinal data; so it is applied to a large sample of cross-sectional data. Indeed a longitudinal approach would be needed as a future research design (and then maybe recall bias could be tackled.)

Methods:

Line 170: why is it necessary to construct an exposure? This is to analyse a condition, isn't it?

In general, the CESD-10 is a validated and well-known instrument, it is not necessary to repeat the items. If you want to keep them, there seems to be one error: (4) I felt that everything you did was an effort – should be everything I did…

Results:

Line 273. Why are there differences in age – didn't you control for it? If higher disadvantaged people are older, then the circumstances might have changed. Moreover, it is a contradiction to Line 288 that older age is associated with lower odds in depr. symptoms

(by the way "reported on Table III" should be "in Table III")

Line 288 again the outcome is not depression but risk of depressive symptoms – I did not check all sentences but strongly recommend to stay precise all over the text

Discussion:

Line 294 – discussion, not discussions

In general there is much literature on inequalities in social capital and depression that could enrich the discussion, especially the question of sense of control. The results of your study show that mobility plays a role as well. I recommend to go more into depth here.

Line 335-37 – A source would be needed for the childhood infections

Limitations: Line 342: Again, LCA is not appropriate to reduce a recall bias but to build subgroups. The recall bias will be a limitation in every survey study. However, a significant limitation is the fact that depression cannot be assessed on the basis of this study, but the presence of depressive symptoms, which is something else. Nonetheless, these people need help.

**Reviewer**	**3**
**Name**	**Kelson, Zoe**
**Affiliation**	**University of Exeter, Mathematics**
**Date**	**27-Mar-2025**
**COI**	**None**

This study aims to examine the long-term effect of childhood socio-economic status (SES) and health on depression among a nationally representative sample of adults in Indonesia.

Reviewer comments:

"Data were obtained from the 2014-2015 Indonesia Family Life Survey (28,279 respondents aged 15 years and older)" [Abstract]

and

"We used data from the Indonesia Family Life Survey (IFLS), a multifunctional longitudinal survey collecting information on individual and household demographic, SES, and health characteristics from about 30,000 respondents from 12,000 households in 13 of 27 provinces in Indonesia...

The IFLS 5 Wave sample contained 36,381 respondents aged 15 and up. We limited our sample to 32,085 respondents aged 18 and over. We then excluded 3,801 respondents who did not have information on their childhood SES and health and 2,925 respondents who did not have information on depression scores. The final sample size was 28,279 people."

Can the authors please comment on whether missing childhood SES and health data can be considered to be missing at random?

Furthermore, can the authors please comment on whether they consider the included sample to be representative for generalisability of the study findings? Can demographics for the analysed cohort as a whole please be tabulated?

Can the authors please clarify the survey design (e.g. were respondents in households from provinces selected using a clustered or stratified randomised sampling design), and confirm if and how this has been adjusted for (e.g. by applying sample weights)?

"The clusters of childhood SES and health were examined using latent class analysis...

Logistic regression models were used to investigate the association between the clusters of childhood circumstances and depression, adjusted for demographics, SES, health behaviour and health status" [Abstract]

and

"In the first step, we derived a latent construct of childhood SES and health, indicating whether the respondents' childhood was in good SES and health conditions....

In the second step, we used logistic regression models to investigate the association between group membership and depression. We performed the analysis in three models. The first model (M1) included our key predictor variable, latent classes of childhood SES and health along with demographics (age and sex) and marital status. The second model (M2) includes current SES (education, social class, wealth, income). The final model (M3) included all those covariates plus health behaviours (smoking behaviours and physical activity)."

The authors have taken an appropriate analytical approach.

Can the authors please consider including family history in the study?

"The sample in each model was reduced to respondents with a full set of observed covariates."

Can the authors please clarify how much data was missing for each covariate?

Can this data be considered to be missing at random?

Can a sensitivity analysis on this complete-case analysis please be conducted, applying multiple imputation for example?

"Table I Latent class analysis for the childhood socio-economic status and early health variables"

Can the authors please consider exploring a LCA with four classes, to confirm that three classes performs the best?

"This study has several limitations. First, the reported results describe associations rather than causations. Considering the nature of observational studies, we cannot determine the causal directions of the observed relationships with certainty. Second, the indicators measuring childhood SES and early health are retrospective self-evaluations, which can lead to issues on measurement errors and exposure misclassification due to recall. To reduce potential recall errors and bias, we used LCA, a data-driven statistical approach on seven indicators of childhood circumstances."

A discussion on the study limitations is provided by the authors.

Can this please be expanded on to address potential residual confounding and the generalisability of the study findings?

Thanks for providing a copy of the STROBE checklist.

VERSION 1 - AUTHOR RESPONSE

Comments from Reviewer 1

This findings of the study suggest the significant association of childhood socioeconomic status with later life depression. However, the authors must address the grammatical issues. For ex.Among the covariates, age, education, 52 wealth quintile, social capital, and depression were associated with depression.

Authors' response

Thank you for the input. We have revised the sentence into (Page 3, lines 58-59):

"Significant covariates included age, education, wealth, and social capital (all p<0.05)."

Comments from Reviewer 1

Further, In table 3, the social capital variable has few numbers. Similarly with other variables Total mobility score, Total activity daily living score and Total instrumental activity daily living score, the numbers are few

Authors' response

We have revised Table 3 to include the mean and standard deviation of the total mobility score, the total activity daily living score, and the total instrumental activity daily living score.

Comments from Reviewer 2

Thank you for the opportunity to review this interesting work. The paper is well-written and structured. It presents data from a survey study that shows the association between socio-economic status and depressive symptoms using latent class analysis.

Few amendments will improve the overall readability and clarity:

Two general questions:

1. Was religion assessed in the survey? If yes, it should be part of the discussion, as it might impact the questions of depressive symptoms to a certain extent.

Authors' response

Thank you for the comments. We have included the variable religion in the analysis.

Comments from Reviewer 2

2. Though socio-economic influences are important to explain mental disorders, there is a big impact of heritability in the prevalence of depression. Did the survey ask for mental health of family members? If not, this limitation should be discussed.

Authors' response

Thank you for highlighting the importance of genetic and familial factors in depression. We agree that heritability plays a significant role. The IFLS includes a variable indicating whether respondents reported psychiatric disorders among family members. We included this variable in our analysis and adjusted it in the regression models. We have also expanded the discussion section to reflect this consideration.

Comments from Reviewer 2

Abstract: The objective focus on depressive symptom not on depression – this applies to several other sentences, as the CES-D 10 does not provide a clinical diagnosis.

Authors' response

We have revised the word "*depression*" to "*depressive symptoms*" throughout the document.

Comments from Reviewer 2

Introduction: First sentence:

- the first source presents data on the increase of depression during COVID in 2020, either you should mention this or look for another, more generalizable source.

Authors' response

We have revised the first sentence by providing a more generalisable source:

"*Depression is among the leading contributors to mental health problems in the world, with a prevalence of around 3588.25 cases per 100,000 population and 46.87 million disability-adjusted life-years (DALYs) reported in 2019*."

Reference: Zhang Y, Jia X, Yang Y, et al. Change in the global burden of depression from 1990-2019 and its prediction for 2030. Journal of Psychiatric Research 2024;178:16-22. doi:

10.1016/j.jpsychires.2024.07.054

Comments from Reviewer 2

- What's more: the interpunctuation of the numbers is confusing 49.9 million can be a dot or a comma, depending on the journals formatting style, but should not be the same as 100.000 (indicating the thousand)

Authors' response

We have revised all numeric punctuation throughout the manuscript to use commas to indicate thousands (e.g., "100,000") in line with standard formatting conventions.

Comments from Reviewer 2

Line 79. Studies have shown that depression can significantly contribute to economic loss, affecting workplace productivity and absenteeism. A significant indirect cost related to productivity loss was also attributed to major depressive disorder [illegible] this seems a repetition, grammar should be checked

Authors' response

Thank you for pointing this out. We have revised the sentence to improve clarity and remove redundancy:

"Studies indicate that depression leads to substantial economic losses through reduced workplace productivity and increased absenteeism."

Comments from Reviewer 2

Line 150: the statement that LCA can reduce recall bias is a somewhat bold claim – LCA is just a statistical method not more. I understand the motivation but don't see that it is necessary to justify the choice in this way. No doubt, that the identification of subgroups is needed.

Authors' response

Thank you for this helpful comment. We acknowledge that our original statement may have overstated the methodological capacity LCA. While LCA does not eliminate recall bias, it allows for identifying subgroups with similar response patterns, which can mitigate some of the noise associated with retrospective self-report data. We have revised the text accordingly to clarify this point (Page 10, Lines 178-181):

"*Latent class analysis (LCA) was used to identify subgroups of individuals based on their patterns of responses across multiple retrospective indicators of childhood socioeconomic status and health. This*

approach provides a classification that may be less sensitive to random errors in individual recall than single-measure analyses."

<u>Comments from Reviewer 2</u>

Line 156: facilitating a more precise analysis of recall bias – this is not the aim of this study. Recall bias is an assumption that might apply to the study population, but cannot be validated on the basis of this data.

<u>Authors' response</u>

We agree that our original wording may have implied an empirical assessment of recall bias, which was not within the scope of our study. We have revised the sentence to clarify that the use of LCA helps manage heterogeneity in self-reported data, rather than directly addressing recall bias (Page 10, Lines 190-193):

"*Through the application of LCA, researchers can classify participants based on shared patterns in their responses to retrospective survey items, providing a structured approach to account for heterogeneity in reported childhood conditions*."

<u>Comments from Reviewer 2</u>

Line 160: applied to longitudinal data – the data analysed was one of the waves of longitudinal data; so it is applied to a large sample of cross-sectional data. Indeed a longitudinal approach would be needed as a future research design (and then maybe recall bias could be tackled.)

<u>Authors' response</u>

Thank you for pointing this out. We agree that the original phrasing may be misleading. Although the Indonesia Family Life Survey is a longitudinal study, our analysis uses a single wave and is cross-sectional in nature. We have revised the sentence accordingly to avoid confusion (Lines 194-198):

"*Therefore, this study aimed to evaluate the impact of childhood SES and health on later-life mental health in Indonesia using latent class analysis applied to cross-sectional data from the 2014/2015 wave of the Indonesia Family Life Survey*."

<u>Comments from Reviewer 2</u>

Methods:

Line 170: why is it necessary to construct an exposure? This is to analyse a condition, isn't it?

<u>Authors' response</u>

Thank you for your thoughtful comment. We agree that "exposure" may not be the most precise term in this context. Our aim was to derive a latent construct representing patterns of childhood socioeconomic and health conditions to be used as the main predictor variable in the regression models. We have revised the text to better reflect this intent (Lines 206-208):

"*We used the fifth wave, which provided retrospective information on childhood conditions, enabling us to derive the primary predictor variable representing early-life socioeconomic status and health through latent class analysis*."

Comments from Reviewer 2

In general, the CESD-10 is a validated and well-known instrument, it is not necessary to repeat the items. If you want to keep them, there seems to be one error: (4) I felt that everything you did was an effort – should be everything I did…

Authors' response

Thank you for this helpful suggestion. We agree that the CES-D-10 is a widely used instrument, and it is not necessary to list all individual items. We have removed the item list and now refer to the CES-D-10 concisely in the revised manuscript:

"*Depressive symptoms were assessed using the 10-item version of the Center for Epidemiologic Studies Depression Scale (CES-D-10), a widely validated self-report instrument. Respondents were asked how often they experienced a range of symptoms during the past week, using a four-point Likert scale (from "rarely or none of the time" to "most of the time"). The two positively worded items were reverse-coded. Scores from the 10 items were summed, with higher scores indicating greater depressive symptom severity. Following established cut-offs, respondents with a total score of ≥10 were classified as having depressive symptoms*."

Comments from Reviewer 2

Results:

Line 273. Why are there differences in age – didn't you control for it? If higher disadvantaged people are older, then the circumstances might have changed. Moreover, it is a contradiction to Line 288 that older age is associated with lower odds in depr. symptoms

Authors' response

Thank you for raising this point. We did not include age as a covariate in the LCA, as the LCA was conducted solely on childhood SES and health indicators to identify subgroups. Age was included as a covariate in the subsequent regression models, which allows us to examine the independent association between the childhood-based latent classes and depression symptoms, controlling for age.

Comments from Reviewer 2

(by the way "reported on Table III" should be "in Table III")

Authors' response

We have revised the words from "reported on Table III" to "in Table III".

Comments from Reviewer 2

Line 288 again the outcome is not depression but risk of depressive symptoms – I did not check all sentences but strongly recommend to stay precise all over the text

Authors' response

We have revised the words "depression" to "depressive symptoms".

Comments from Reviewer 2

Discussion:

Line 294 – discussion, not discussions

Authors' response

We have revised the word "discussion" to "discussions".

Comments from Reviewer 2

In general there is much literature on inequalities in social capital and depression that could enrich the discussion, especially the question of sense of control. The results of your study show that mobility plays a role as well. I recommend to go more into depth here.

Authors' response

We have expanded the discussion section to more thoroughly explore how social capital and sense of control mediate the relationship between childhood socio-economic status and depression in the Discussion section:

"Our findings also suggest that social capital and a sense of control may be key mechanisms through which early life disadvantage translates into adult mental health outcomes. Individuals from low-SES backgrounds often have reduced access to supportive social networks and fewer opportunities to develop a sense of mastery or autonomy. This diminished social capital can limit their coping strategies and increase vulnerability to depression. Prior research has shown that individuals with a strong sense of control and social support tend to experience fewer depressive symptoms, even in the face of economic or environmental adversity.[30 31] Conversely, those with limited control over their life circumstances are more likely to experience chronic stress, which is a well-established pathway to depression.[31 32] The lower social capital scores observed among respondents with high early life disadvantage in our study may therefore serve as both a marker and mediator of depression risk.

In addition, our results showed that functional limitations, specifically, reduced mobility, are significantly associated with increased odds of depression. Mobility impairments can restrict daily activities, reduce social engagement, and foster dependency, all of which can contribute to emotional distress and poor mental health.[33] This finding aligns with existing literature that highlights the reciprocal relationship between physical disability and mental health.[34 35] Not only can reduced mobility lead to isolation and a loss of autonomy, but depression itself can also exacerbate physical symptoms and limit motivation to remain active. Therefore, interventions that promote physical functioning and social participation may serve a dual role in mitigating depression, particularly among individuals with early-life disadvantages."

Comments from Reviewer 2

Line 335-37 – A source would be needed for the childhood infections

Authors' response

We have added the reference for the childhood infections:

33. Chaplin AB, Jones PB, Khandaker GM. Association between common early-childhood infection and subsequent depressive symptoms and psychotic experiences in adolescence: A population-based longitudinal birth cohort study. Psychological Medicine 2020 doi: 10.1017/s0033291720004080.

Comments from Reviewer 2

Limitations: Line 342: Again, LCA is not appropriate to reduce a recall bias but to build subgroups. The recall bias will be a limitation in every survey study. However, a significant limitation is the fact that depression cannot be assessed on the basis of this study, but the presence of depressive symptoms, which is something else. Nonetheless, these people need help.

Authors' response

We have added this point in our Discussion and revised the Limitation section to:

"*Second, the retrospective assessment of childhood SES and health indicators may be subject to recall bias, a limitation inherent to all survey studies using self-reported life course data. While LCA helped identify meaningful subgroups based on response patterns across multiple indicators, this methodological approach does not eliminate fundamental recall bias.* "

Comments from Reviewer 3

This study aims to examine the long-term effect of childhood socio-economic status (SES) and health on depression among a nationally representative sample of adults in Indonesia.

"Data were obtained from the 2014-2015 Indonesia Family Life Survey (28,279 respondents aged 15 years and older)" [Abstract]

and

"We used data from the Indonesia Family Life Survey (IFLS), a multifunctional longitudinal survey collecting information on individual and household demographic, SES, and health characteristics from about 30,000 respondents from 12,000 households in 13 of 27 provinces in Indonesia...

The IFLS 5 Wave sample contained 36,381 respondents aged 15 and up. We limited our sample to 32,085 respondents aged 18 and over. We then excluded 3,801 respondents who did not have information on their childhood SES and health and 2,925 respondents who did not have information on depression scores. The final sample size was 28,279 people."

Can the authors please comment on whether missing childhood SES and health data can be considered to be missing at random?

Authors' response

Thank you for this important comment. To assess whether the missing data in our study could be considered Missing Completely At Random (MCAR), we conducted Little's MCAR test in Stata using all variables with missing values, including childhood SES and health indicators, depression status, health conditions, and relevant covariates. The results reject the null hypothesis that data are missing completely at random ($p<0.001$), indicating that the missingness is not MCAR and may depend on observed characteristics. To address this issue and to reduce the risk of bias introduced by complete case analysis, we addressed the missing data using multiple imputations (Supplementary Table 2). The results from the imputed models were consistent with our complete case analysis, reinforcing the robustness of the associations found between early-life disadvantage and adult depression. To reflect this in the manuscript, we have added the following to the Discussion section:

"*We used multiple imputation with chained equations for our regression models. The imputed results were consistent with the complete case analysis, suggesting our findings are robust despite potential data limitations.*"

Comments from Reviewer 3

Furthermore, can the authors please comment on whether they consider the included sample to be representative for generalisability of the study findings? Can demographics for the analysed cohort as a whole please be tabulated?

Authors' response

We have provided a detailed comparison of demographic characteristics between the total IFLS Wave 5 sample, the included (analytic) sample, and excluded participants in Supplementary Table 1. The Indonesia Family Life Survey (IFLS) is designed to be nationally representative of 83% of the Indonesian population, covering 13 of the country's 27 provinces across all major regions. Our analytic sample of 29,140 respondents, after applying inclusion criteria and excluding those with missing childhood data. remains large and diverse, maintaining the geographical and demographic spread of the original IFLS design.

While we observed some differences between included and excluded respondents (e.g., lower female representation and younger average age among excluded cases), the included sample still broadly reflects the demographic structure of the IFLS population. We acknowledge these differences as a limitation, which we now note in the Discussion section, but we consider the sample sufficiently representative to support cautious generalisation of the findings to the broader adult population in Indonesia.

Comments from Reviewer 3

Can the authors please clarify the survey design (e.g. were respondents in households from provinces selected using a clustered or stratified randomised sampling design), and confirm if and how this has been adjusted for (e.g. by applying sample weights)?

Authors' response

Thank you for your comment. The IFLS uses a multistage stratified sampling design, with stratification at the provincial level and clustering at the community and household levels. In our analysis, we have applied individual-level sampling weights provided by the RAND Corporation to ensure national

representativeness. We also adjusted for clustering at the primary sampling unit level by using robust standard errors. This information has been added to the Methods section.

We have added these sentences in the Methods section:

"*The IFLS employs a multistage stratified sampling design, with stratification by province and clustering at the community and household levels. We applied sampling weights to account for this complex design and used robust standard errors clustered at the primary sampling unit level in all analyses*."

<u>Comments from Reviewer 3</u>

"The clusters of childhood SES and health were examined using latent class analysis...

Logistic regression models were used to investigate the association between the clusters of childhood circumstances and depression, adjusted for demographics, SES, health behaviour and health status" [Abstract]

and

"In the first step, we derived a latent construct of childhood SES and health, indicating whether the respondents' childhood was in good SES and health conditions....

In the second step, we used logistic regression models to investigate the association between group membership and depression. We performed the analysis in three models. The first model (M1) included our key predictor variable, latent classes of childhood SES and health along with demographics (age and sex) and marital status. The second model (M2) includes current SES (education, social class, wealth, income). The final model (M3) included all those covariates plus health behaviours (smoking behaviours and physical activity)."

The authors have taken an appropriate analytical approach.

Can the authors please consider including family history in the study?

<u>Authors' response</u>

We agree that the family history of mental health is an important potential confounder. We have included a variable indicating self-reported family history of psychiatric disorders in our regression models. This was added as an additional covariate in Model 3. We have also updated the manuscript to reflect this inclusion.

Despite the low frequency of this variable (75 responded yes to this question), the logistic regression model (model 3) indicates a statistically significant and positive association between family history and depression. This suggests that, within our sample, individuals reporting a family history of psychiatric disorders have a significantly higher odds of experiencing depression compared to those without such a history.

<u>Comments from Reviewer 3</u>

"The sample in each model was reduced to respondents with a full set of observed covariates."

Can the authors please clarify how much data was missing for each covariate?

Authors' response

We have now included a detailed breakdown of the extent of missing data for each covariate used in the regression models. This information is presented in Table 3, which shows both the number and percentage of missing observations for each variable included in the analysis.

Comments from Reviewer 3

Can this data be considered to be missing at random?

Authors' response

Thank you for this important follow-up question. To assess the missing data mechanism, we conducted Little's MCAR test across all variables with missing values used in the analysis, including childhood SES and health indicators, depressive symptoms, and all covariates. This result rejects the null hypothesis that the data are Missing Completely At Random (MCAR), suggesting that missingness is related to observed characteristics in the dataset. While we cannot fully confirm whether the data meet the assumptions for being Missing At Random (MAR), the use of multiple imputation with a rich set of auxiliary variables, such as demographic, socio-economic, and health indicators, helps to make the MAR assumption more plausible. We have therefore applied multiple imputation in our sensitivity analysis, and the imputed results were consistent with those from the complete-case analysis, supporting the robustness of our findings. This has been noted in the revised Methods and Discussion sections, and the imputation results are provided in Supplementary Table 2.

Comments from Reviewer 3

Can a sensitivity analysis on this complete-case analysis please be conducted, applying multiple imputation for example?

Authors' response

Thank you for this constructive suggestion. In response, we have conducted a sensitivity analysis using multiple imputation to address potential bias from missing data in our complete-case analysis. We used multiple imputation by chained equations (MICE), incorporating all variables used in the logistic regression models. This includes childhood SES and health indicators, depressive symptoms, and relevant demographic and health covariates. The results from the imputed models are presented in Supplementary Table 2. They are consistent with the findings from the complete-case analysis, showing similar direction and magnitude of associations between early-life disadvantage and depression in adulthood. This strengthens our confidence in the robustness of the main findings. To ensure transparency, we have also referenced this sensitivity analysis in the Methods and Discussion sections of the revised manuscript.

Comments from Reviewer 3

"Table I Latent class analysis for the childhood socio-economic status and early health variables"

Can the authors please consider exploring a LCA with four classes, to confirm that three classes performs the best?

Authors' response

We also explored a four-class solution, but the model did not converge despite extended iterations, suggesting poor model stability. Therefore, we retained the three-class model based on better convergence, fit statistics (lower AIC and BIC), and interpretability. We have provided the model fit of each model in Table 1.

Comments from Reviewer 3

"This study has several limitations. First, the reported results describe associations rather than causations. Considering the nature of observational studies, we cannot determine the causal directions of the observed relationships with certainty. Second, the indicators measuring childhood SES and early health are retrospective self-evaluations, which can lead to issues on measurement errors and exposure misclassification due to recall. To reduce potential recall errors and bias, we used LCA, a data-driven statistical approach on seven indicators of childhood circumstances."

A discussion on the study limitations is provided by the authors.

Can this please be expanded on to address potential residual confounding and the generalisability of the study findings?

Authors' response

Thank you for this valuable suggestion. We have expanded the limitations section to include a discussion of potential residual confounding due to unmeasured or imperfectly measured variables, and to reflect on the generalisability of our findings within and beyond the Indonesian context. These additions have been incorporated into the revised manuscript:

"*This study has several limitations. First, the reported results describe associations rather than causation. As with any observational study, we cannot determine the direction of the observed relationships with certainty. Second, the indicators measuring childhood SES and early health were based on retrospective self-reports, which are subject to recall bias and potential misclassification. While we used latent class analysis (LCA) to identify patterns in self-reported data, this approach does not eliminate recall bias. Third, although we adjusted for a wide range of demographic, socioeconomic, and health-related covariates, the possibility of residual confounding from unmeasured factors, such as early-life parental mental health or community-level exposures, cannot be ruled out. Lastly, while the IFLS is nationally representative of the 13 provinces included, findings may not be fully generalisable to provinces not covered in the survey or to other low- and middle-income country contexts without similar demographic or socioeconomic profiles.*"

VERSION 2 - REVIEW

**Reviewer**	**3**
**Name**	**Kelson, Zoe**
**Affiliation**	**University of Exeter, Mathematics**
**Date**	**03-Jun-2025**

COI

Many thanks to the authors for their responses and revisions. The additional exploration of missing data, introduction of sensitivity analyses, and including family history in model 3, have all substantially strengthened the reporting of this study.
